# Performance and Accuracy of Four Open-Source Tools for *In Silico* Serotyping of *Salmonella* spp. Based on Whole-Genome Short-Read Sequencing Data

**DOI:** 10.1128/AEM.02265-19

**Published:** 2020-02-18

**Authors:** Laura Uelze, Maria Borowiak, Carlus Deneke, István Szabó, Jennie Fischer, Simon H. Tausch, Burkhard Malorny

**Affiliations:** a German Federal Institute for Risk Assessment (BfR), Berlin, Germany; University of Naples Federico II

**Keywords:** serotyping, *Salmonella*, O antigen, whole-genome sequencing, serovar prediction

## Abstract

We compared the performance of four open-source *in silico Salmonella* typing tools (SeqSero, SeqSero2, *Salmonella In Silico* Typing Resource [SISTR], and Metric Oriented Sequence Typer [MOST]) to assess their potential for replacing laboratory serological testing with serovar predictions from whole-genome sequencing data. We conducted a retrospective analysis of 1,624 *Salmonella* isolates of 72 serovars submitted to the German National Salmonella Reference Laboratory between 1999 and 2019. All isolates are derived from animal and foodstuff origins. We conducted Illumina short-read sequencing and compared the *in silico* serovar prediction results with the results of routine laboratory serotyping. We found the best-performing *in silico* serovar prediction tool to be SISTR, with 94% correctly typed isolates, followed by SeqSero2 (87%), SeqSero (81%), and MOST (79%). Furthermore, we found that mapping-based tools like SeqSero and SeqSero2 (allele mode) were more reliable for the prediction of monophasic variants, while sequence type and cluster-based methods like MOST and SISTR (core-genome multilocus sequence type [cgMLST]), showed greater resilience when confronted with GC-biased sequencing data. We showed that the choice of library preparation kit could substantially affect O antigen detection, due to the low GC content of the *wzx* and *wzy* genes. Although the accuracy of computational serovar predictions is still not quite on par with traditional serotyping by *Salmonella* reference laboratories, the command-line tools investigated in this study perform a rapid, efficient, inexpensive, and reproducible analysis, which can be integrated into in-house characterization pipelines. Based on our results, we find SISTR most suitable for automated, routine serotyping for public health surveillance of *Salmonella*.

**IMPORTANCE** *Salmonella* spp. are important foodborne pathogens. To reduce the number of infected patients, it is essential to understand which subtypes of the bacteria cause disease outbreaks. Traditionally, characterization of *Salmonella* requires serological testing, a laboratory method by which *Salmonella* isolates can be classified into over 2,600 distinct subtypes, called serovars. Due to recent advances in whole-genome sequencing, many tools have been developed to replace traditional testing methods with computational analysis of genome sequences. It is crucial to validate that these tools, many already in use for routine surveillance, deliver accurate and reliable serovar information. In this study, we set out to compare which of the currently available open-source command-line tools is most suitable to replace serological testing. A thorough evaluation of the differing computational approaches is highly important to ensure the backward compatibility of serotyping data and to maintain comparability between laboratories.

## Abstract (precis)

*Salmonella* spp. are important foodborne pathogens. To reduce the number of infected patients, it is essential to understand which subtypes of the bacteria cause disease outbreaks. Traditionally, characterization of *Salmonella* requires serological testing, a laboratory method by which *Salmonella* isolates can be classified into over 2,600 distinct subtypes, called serovars. Due to recent advances in whole-genome sequencing, many tools have been developed to replace traditional testing methods with computational analysis of genome sequences. It is crucial to validate that these tools, many already in use for routine surveillance, deliver accurate and reliable serovar information. In this study, we set out to compare which of the currently available open-source command-line tools is most suitable to replace serological testing. A thorough evaluation of the differing computational approaches is highly important to ensure the backward compatibility of serotyping data and to maintain comparability between laboratories.

## INTRODUCTION

Since the 1960s, *Salmonella* species isolates have been differentiated into serovars, e.g., Salmonella enterica serovar Enteritidis or Typhimurium. This distinction has proven extremely useful, since characteristics like host specificity, virulence, and pathogenicity usually correlate well with serovar assignments. For example, one of the most commonly isolated *Salmonella* serovars worldwide is S. enterica serovar Enteritidis, which is often found in contaminated eggs and can cause salmonellosis in humans (1).

Consequently, serovar assignment has provided scientists, public health experts, veterinarians, and the general public with an effective terminology which has since shaped the core of any *Salmonella* monitoring and surveillance scheme. Interestingly, the process of serotyping does not take markers of virulence or pathogenicity into account directly but instead is based on natural variations in two cell surface structures. These two cell surface structures are the O antigen, a cell surface protein, and the H antigen, which forms part of the flagella. *Salmonella* spp. feature a wide variety of these flagellar H and lipopolysaccharide O antigens. Serotyping utilizes these variations by assigning a number and letter code to each known H and O antigen, followed by classifying different combinations according to the White-Kauffmann-Le Minor scheme (2). Currently, the White-Kauffmann-Le Minor scheme denotes more than 2,600 *Salmonella* serotypes. Besides assigning a specific antigenic formula, the typing scheme also denotes unique serotype names, which often point to the geographical origin of the first isolate investigated. Traditionally, serotype names have only been allocated to isolates belonging to S. enterica subsp. *enterica*, while serotypes of other subspecies are denoted solely by their antigenic formula, such as serovar 61:k:1,5,(7) of S. enterica subsp. *diarizonae*.

The serotyping process in the laboratory involves a series of serological tests, in which the presence of a specific O antigen or H antigen is verified through agglutination between the cells and specific antisera. No expensive equipment is required for serological testing, and for this reason, laboratory serotyping has become a well-established gold standard method. However, there are several disadvantages to laboratory serotyping. Most importantly, agglutination tests can only detect O and H antigens that are currently expressed by the cell. In order to induce the expression of all possible flagellar H antigens (2nd-phase H antigen), isolates need to be passaged through different media, which can be a labor-intensive and time-consuming process. Second, the large number of known serovars makes it impractical for laboratories to keep all antisera necessary for typing of rare serotypes in stock. Another issue is that, although conducting serological tests is relatively easy, producing the antisera is not and requires stringent quality control to prevent false-positive results. To overcome these drawbacks, several *in silico* methods have been developed in recent years to replace traditional serotyping. Among the first implemented is the detection of sequence differences that cause the variations in either the O or the H antigen. Although there is no single gene encoding either the O or the H antigen, it is known that the two flagellar antigens are encoded by the *fliC* and *fljB* gene, respectively, and that the O lipopolysaccharide is encoded by the O antigen flippase (*wzx*) and polymerase (*wzy*) (*rfb* gene cluster) genes. Sequencing data derived from whole-genome-sequencing (WGS) provides the perfect basis for an analysis of these gene sequences for predicting the serovar. Several tools have been introduced that are able to infer serovar predictions from the analysis of O and H antigen sequences derived from whole-genome sequencing data. Among these tools currently available are SeqSero (3), as well as its successor version, SeqSero2 (4), and the *Salmonella In Silico* Typing Resource (SISTR) (5). All three tools implement a mapping step, which aligns the sequencing reads or the assembled genome to a reference database of O and H antigen allele sequences and then assigns the antigenic formula and/or the serovar name based on the best-scoring alignments. In addition, SeqSero2 is capable of breaking reads into k-mers, which are then compared to the frequency of unique k-mers of serotype determinants. Another interesting approach for *in silico* serovar prediction is to infer serovars from multilocus sequence types, which have been shown to correlate well with *Salmonella* serovars. For example, isolates of S. enterica serovar Mbandaka usually have sequence type 413, based on the 7-gene multilocus sequence typing (MLST) scheme (6, 7). Among the tools implementing this method are Metric Oriented Sequence Typer (MOST) (8), which infers serovars from MLST, and SISTR (5), which can predict serovars based on phylogenetic clustering of core genome MLST (cgMLST) or based on a k-mer reference search (mash). Several studies have further investigated the utility of lineage-specific gene markers for the identification of polyphyletic serovars (9–11). However, we are not aware of any currently publicly available program that implements the findings from these studies.

A number of benchmarking studies have been conducted to evaluate the performance of different *in silico* serotyping tools. The majority of studies were conducted by developers of the tools, mostly with their own unique set of samples. For example, MOST was validated with ∼6,900 isolates of human origin submitted to the Salmonella Reference Service at Public Health England (PHE) (12); SeqSero was validated with raw reads from genomes of ∼300 *Salmonella* isolates with known serovars and ∼3,700 isolates from public databases (3), as well as in a second independent study with ∼1,000 isolates from the laboratory inventory collected by the U.S. Food and Drug Administration (13); its successor SeqSero2 was validated recently with ∼2,300 isolates submitted to the National Antimicrobial Resistance Monitoring System at the U.S. Centers for Disease Control and Prevention (4), while its improved performance over that of SeqSero was benchmarked on the same sample set as in Zhang et al. (3); and the SISTR tool was validated with 42,400 isolates from public databases (14), as well as with ∼4,200 publicly available sequences (5). Yachison and colleagues (15) compared the performance of SeqSero to that of SISTR on 813 serotyped isolates from Canada, while a comparison of the performance of SeqSero2 to that of SISTR was conducted during the validation of SeqSero2 (4).

To date, the most comprehensive, independent comparative study was published in the 2018 ENGAGE report (16). In that study, 786 serotyped broad-range isolates were analyzed with the tools SeqSero, SISTR, MOST, and SalmonellaTypeFinder (SalmonellaTypeFinder is based on SeqSero2 and MLST). The study found that SISTR achieved the highest correlation with conventional serotyping (88%), followed by SalmonellaTypeFinder (85%), MOST (85%), and SeqSero (65%). However, in that study, none of the isolates were retested to ensure correct serotyping. Furthermore, although web-based online interfaces, such as the SISTR webtool (https://lfz.corefacility.ca/sistr-app/) used in the ENGAGE study, make *in silico* serotyping tools easily and widely accessible, they are not suitable for generating high-throughput, independent, reliable and reproducible results. Only their command-line program versions can be integrated into in-house bacterial characterization analysis pipelines, which allow rapid, efficient, customized, controlled, and reproducible bioinformatic analysis of WGS data on a day-to-day basis.

For this reason, we set out to analyze and compare the performance of four tools, MOST, SeqSero, SeqSero2 (k-mer and allele mode), and SISTR (all subresults), in their command-line versions, for *in silico Salmonella* serovar prediction based on trimmed raw reads and assembled Illumina short-read sequences obtained from our laboratory inventory of 1,624 *Salmonella* species isolates from nonhuman origins.

## RESULTS

### Comparison of tool performance.

**FIG 1:**
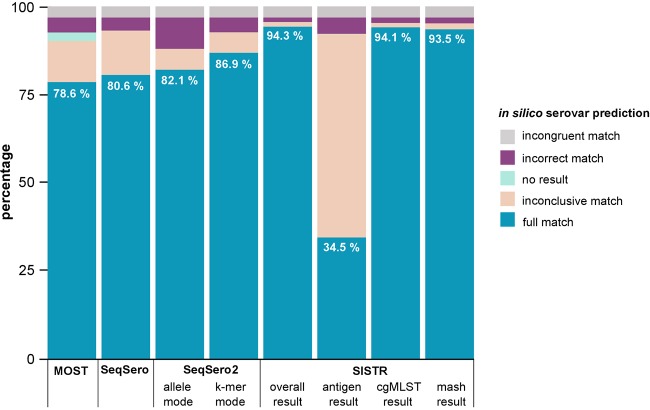
Graphical representation of the *in silico* typing results by tool. *In silico* serotyping results were compared to laboratory serological testing and categorized as full, inconclusive, incongruent, or incorrect matches in keeping with the methodology of Yachison and colleagues (15). The stacked-bar chart shows the results summarized by tool and/or subresult. The percentages of correct results per tool are shown.

**(i) Correct serotype predictions overall.** The serovars of about two-thirds of all isolates were correctly and unambiguously predicted by all four tools (1,057 isolates [65%]). Individually, the best-performing *in silico* serovar prediction tool was SISTR (overall result), with 94% correctly typed isolates, followed by SeqSero2 (k-mer mode) (87%), SeqSero2 (allele mode) (82%), SeqSero (81%), and MOST (79%). A graphical representation of the comparison is shown in Fig. 1, while exact percentages are given in Table 1. An extensive overview of tool performance grouped by serovar is given in Table S2 in the supplemental material.

**TABLE 1:** Overview of tool performance

Serovar prediction result	% of isolates with result using:							
	SeqSero	SeqSero2		SISTR				MOST
		k-mer	Allele	Overall	Antigen	cgMLST	mash	
Full	80.6	86.88	82.08	94.27	34.54	94.09	93.53	78.57
Inconclusive	12.56	5.79	5.91	1.23	57.64	1.23	1.66	11.64
Incongruent	3.14	3.14	3.14	3.14	3.14	3.14	3.14	3.14
Incorrect	3.69	4.19	8.87	1.29	4.62	1.54	1.66	4.25
No result	0	0	0	0.06	0.06	0	0	2.4

**(ii) Incorrect results.** About 10% of all isolates were serotyped with an incorrect result by at least one tool. SISTR produced the fewest incorrectly typed isolates, with 21 isolates in total. In contrast, MOST, SeqSero, and SeqSero2 each typed a considerable number, about 4% of all isolates, incorrectly. The majority of serovars wrongly reported by SeqSero and SeqSero2 could be traced to a missing somatic O antigen. Both SeqSero and SeqSero2 were consistently unable to determine an O antigen for a number of S. enterica serovars, such as Agona, Barelly, Derby, Enteritidis, Infantis, Mbandaka, Paratyphi B var. Java, Typhimurium, and Virchow, as shown by the results in Table 2. Interestingly, although SeqSero2 performed better than SeqSero overall, it produced a slightly higher number of incorrect results. These could mainly be traced back to SeqSero2 classifying a large number of S. enterica serovar Enteritidis (1,9,12:g,m:−) isolates wrongly as S. enterica serovar Hillingdon (9,46:g,m:−:−) in k-mer mode, an error that was not observed in allele mode.

**TABLE 2:** Serovar prediction for isolates with missing O antigen

Serovar	Seroformula	Serotyping result using SeqSero/SeqSero2
Agona	1,4,[5],12:f,g,s:[1,2]	−:f,g,s:−
Bareilly	6,7,14:y:1,5	−:y:1,5
Derby	1,4,[5],12:f,g:[1,2]	−:f,g:−
Enteritidis	1,9,12:g,m:−	−:g,m:−
Infantis	6,7,14:r:1,5	−:r:1,5
Mbandaka	6,7,14:z10:e,n,z15	−:z10:e,n,z15
Paratyphi B var. Java	1,4,[5],12:b:1,2	−:b:1,2
Virchow	6,7,14:r:1,2	−:r:1,2
Typhimurium	1,4,[5],12:i:1,2	−:i:1,2

MOST was unable to resolve serovars from subspecies other than S. enterica subsp. I and classified S. enterica subsp. IIIb 61:k:1,5,7 as S. enterica subsp. *arizonae*, thereby producing a large number of incorrect results (54 of the S. enterica subsp. IIIb isolates in total), as shown by the examples in Table S3.

**(iii) Inconclusive results.** Not taking into account inconclusive results caused by an incorrect differentiation into monophasic (1,4,[5],12:i:−) and biphasic S. enterica serovar Typhimurium, about 11% of isolates received an inconclusive result from either SeqSero or SeqSero2, while SISTR and MOST did not produce any inconclusive results. Overall, SeqSero produced the highest number of inconclusive results. Typical serovars that SeqSero was unable to resolve were S. enterica serovars Enteritidis (typed as Enteritidis or Gallinarum), Hadar (typed as Hadar or Istanbul), Indiana (typed as Indiana or II 4,12:z:1,7), Kottbus (typed as Kottbus or Ferruch), and Senftenberg (typed as Senftenberg or Dessau). Its successor version, SeqSero2, featured fewer inconclusive results, both in allele and in k-mode. However, like SeqSero, SeqSero2 was unable to resolve S. enterica serovars Enteritidis, Kottbus, and Senftenberg.

**(iv) No results.** Between 6 and 8% of all isolates could not be typed with a serovar name by SeqSero or SeqSero2. However, similarly to SISTR, in addition to the serovar name, SeqSero and SeqSero2 also output the individual O, H1, and H2 antigen assignments, making it possible to infer a seroformula in cases where no serovar name could be determined. Taking the seroformula into account, 99% of all isolates (including those which were nontypeable in the laboratory) could be assigned a serovar name or seroformula through *in silico* serovar prediction. Only MOST was unable to assign a sequence type and, therewith, a serovar to a number of isolates (47 in total).

**(v) Incongruent results.** The *in silico* typing tools were able to successfully predict serovars for isolates for which no laboratory serotyping result could be obtained. These isolates, characterized as “rough” or “nonmotile,” made up 3.1% of the total sample set (51 isolates in total). A serovar could be determined for all 51 isolates by the *in silico* serovar prediction tools with consistent results for most isolates across the 4 different tools. Over half of the rough/nonmotile isolates were typed as S. enterica serovar Typhimurium, Typhimurium monophasic, or Enteritidis, while other isolates were determined to be of S. enterica serovars Choleraesuis, Derby, Fulica, Infantis, Ohio, Paratyphi B var. Java, and Livingstone.

**(vi) Statistical significance.** Fisher’s exact test was used to evaluate the statistical significance of differences between the successful predictions of each tool. A *P* value of less than or equal to 0.05 was considered statistically significant. Incongruent results were removed from the statistical analyses, and correctly typed serovar results were compared to the sum of all inconclusive and incorrectly typed isolates. An overview of the pairwise-calculated *P* values is shown in Table S4. *P* values were greater than or equal to 0.05 for all possible pairwise combinations between SeqSero, SeqSero2 (both allele and k-mer mode), and MOST. Therefore, SeqSero, SeqSero2, and MOST produce statistically comparable results. The overall SISTR result, the SISTR mash result, and the SISTR cgMLST result were significantly better than the results of SeqSero, SeqSero2 in allele mode, and MOST. Only the SeqSero2 k-mer result was comparable with the SISTR overall, mash, and cgMLST results. Across the SISTR results, all result types were comparable, with the exception of SISTR antigen, which performed significantly worse than all SISTR subresults. In conclusion, SISTR (overall, cgMLST, and mash result) and SeqSero2 in k-mer mode show significantly better performance than all other tools.

### Analysis of shortcomings and issues.

**(i) Antigen-mapping-based methods are more suitable for the detection of monophasic variants.** The serovar Typhimurium often occurs in its monophasic variant, which lacks the second flagellar phase (seroformula 1,4,[5],12:i:−). Together, mono- and biphasic Typhimurium isolates made up ∼25% of the complete sample set used in this study. While MOST is unable to identify the monophasic variant, SeqSero and SeqSero2 are capable of detecting monophasic Typhimurium as “potential monophasic variant of Typhimurium.” Similarly, SISTR is able to recognize the monophasic variation with its seroformula 4,[5],12:i:−. While a majority of all Typhimurium isolates (>90%) were correctly identified as either mono- or biphasic in concordance with the laboratory serotyping result by all tools except MOST, about 32 isolates received a divergent result by at least one tool. These erroneous results could be classified into the false-positive detection of the second flagellum antigen (monophasic laboratory result and biphasic *in silico* typing result) and into the false-false failure to detect the second antigen when laboratory serotyping verified its presence (biphasic laboratory result and monophasic *in silico* typing result). Interestingly, no false-false errors could be attributed to any of the antigen-mapping-based methods as implemented in SeqSero, SeqSero2, and SISTR antigen, which reliably detected the presence of the second H antigen. In contrast, non-mapping-based methods, such as SISTR mash and cgMLST, wrongly classified a number of biphasic Typhimurium isolates (∼10 isolates) as monophasic variants. These erroneous assignments are caused by the high overall similarity of these isolates to a monophasic reference genome with the antigenic formula I 4,[5],12:i:−, as was also reported by Yachison and colleagues (15). This error is of particular importance given that SISTR assigns the cgMLST result as the overall serotyping result whenever the cgMLST distance from the closest reference genome is very small (less than 0.05). Of course, the same mechanism potentially causes the classification of monophasic isolates with a small cgMLST distance from a biphasic reference genome as biphasic, as was observed in a few cases in this study (e.g., isolate 14-SA01066). False-positive results in which mapping-based tools predicted the presence of the second flagellum antigen but laboratory testing determined the monophasic variant can be attributed to the fact that this antigen is not always expressed and, therefore, it is often undetectable in the laboratory. This was verified by confirming the presence of the second H antigen in the genome sequence through BLAST for about 15 isolates. For a small number of these, BLAST revealed a truncated H antigen of ∼500 bp (the full gene length is ∼1,500 bp).

**(ii) GC bias negatively affects O antigen recognition.** SeqSero and SeqSero2 failed to determine the O antigen for a considerable number of isolates for which the O antigen could be detected through serological laboratory testing. Isolates were not equally affected throughout the sample set, and the problem mainly affected the serovar prediction of nine S. enterica serovars that can be divided into three groups by their O antigen: group O:4 (B): 1,4,[5] (serovars Agona, Derby, Paratyphi B var. Java, and Typhimurium); group O:7 (C1): 6,7,14 (serovars Bareilly, Infantis, Mbandaka, and Virchow), and group O:9 (D1): 1,9,12 (serovar Enteritidis), as shown in Table 2.

SeqSero2 in allele mode was most susceptible to missed O antigens and failed to recognize the O antigen for a total of 113 isolates. Of those isolates, SeqSero was unable to determine the O antigen for 41 isolates, while SeqSero2 in k-mer mode was more successful, only missing the O antigen for 21 isolates. Due to the fact that MOST exclusively and SISTR optionally infer the serovar from sequence types, missed O antigens were not apparent from the SISTR and MOST overall prediction results. However, SISTR alerts the user with a warning whenever it is unable to determine the *wzx/wzy* genes through H antigen mapping. All three tools (SISTR, SeqSero, and SeqSero2) perform a nucleotide BLAST search for the *wzx*/*wzy* genes, utilizing the result of whichever gene gives the best (highest-scoring) BLAST result. From the SISTR documentation, it is clear that if both give an equally good match, then the result from the *wzx* gene is preferred. Over the complete sample set, SISTR classified 312 isolates (19.2%) as missing the *wzx/wzy* gene sequences. Unlike SeqSero and SeqSero2, SISTR does not assign the antigen from the highest scoring BLAST result but instead determines the respective serogroup, from which it then infers the O antigen through a lookup table. Therefore, whenever SISTR is unable to detect the O antigen sequence through alignment of contigs, it produces an antigen result with all possible serogroup combinations, leading to the observed high number of inconclusive results for the SISTR antigen subresult.

Zhang and colleagues (3) first observed isolates with no or few reads with homology to the *rfb* cluster while validating the performance of SeqSero, but the study did not find a mechanistic explanation for this artifact. Later, Yachison and colleagues (15), who also noted the failure of O antigen recognition, reported that the O antigen locus features increased fragmentation, which together with a single size selection for insert sizes above a 500-bp minimum, removes the smaller fragments of the O antigen locus from the genomic library (generated with the Nextera XT DNA library preparation kit in this study), thus removing these sequences from the final sequencing data. According to Yachison et al. (15), this causes the *rfb* region to be split over two contigs during genome assembly, thus impeding *rfb* cluster recognition by *in silico* tools (in the study by Yachison et al. [15], both SISTR and SeqSero were run on assembled draft genome sequences).

Of the sample set used in our study, more than half of all isolates (59%, *n* = 955) were processed with the Nextera XT DNA library preparation kit (XT kit), while the remaining 41% of isolates (*n* = 669) were processed with the Nextera DNA Flex library preparation kit (Flex kit). Thus, our sample set afforded us an opportunity to compare the effect of the choice of library preparation kit on the number of incorrect results.

**FIG 2:**
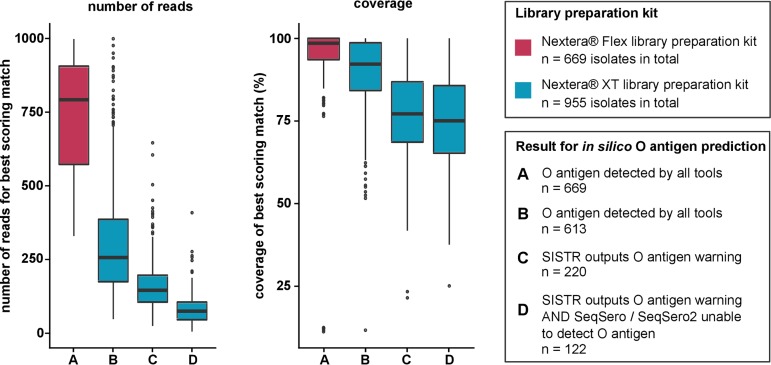
Quality parameters of reads mapped against the O antigen sequence. Trimmed reads of all 1,624 isolates were mapped with SRST2 (27) against the O antigen sequence database of SISTR (containing both the *wzx* and the *wzy* gene sequences). Only the best-scoring match was considered for each isolate. Quality parameters (number of reads mapped and percent coverage) for all respective best-scoring matches were extracted, statistically evaluated, and visualized in box plots. Results are divided into four categories (A to D) depending on whether the *in silico* serotyping tools could successfully determine the O antigen from the sequencing data and which library kit was used; the fill colors indicate the library kit with which the respective isolates were sequenced (Flex kit, Nextera DNA Flex library preparation kit; XT kit, Nextera XT DNA library preparation kit [both Illumina]). The number of isolates per category is given in the key.

We noted that all isolates for which the O antigen could not be determined by SeqSero or SeqSero2 and those which were flagged by SISTR with the missing O antigen warning were processed with the XT kit (Table 3). We subsequently analyzed the number and coverage of trimmed reads mapped against the SISTR O antigen sequence database and found that sequencing data derived from isolates prepared with the XT kit contained fewer reads with lower coverage of the *rfb* cluster, as shown in Fig. 2.

**TABLE 3:** Comparison of the numbers of isolates of different S. enterica serovars for which *in silico* serotyping tools failed to determine the O antigen and the library preparation kits with which isolates were sequenced

O antigen detection result	Serovar	Library kit^*a*^	No. of isolates with result^*b*^
Detected	Agona	Flex	28
	Agona	XT	24
	Bareilly	Flex	1
	Bareilly	XT	1
	Enteritidis	Flex	236
	Enteritidis	XT	71
	Infantis	Flex	46
	Infantis	XT	40
	Mbandaka	Flex	4
	Mbandaka	XT	28
	Paratyphi B var. Java	Flex	11
	Paratyphi B var. Java	XT	66
	Typhimurium	Flex	48
	Typhimurium	XT	135
	Typhimurium monophasic (1,4,[5],12:i:−)	Flex	53
	Typhimurium monophasic (1,4,[5],12:i:−)	XT	115
SISTR O antigen detection warning^*c*^	Agona	XT	11
	Enteritidis	XT	53
	Infantis	XT	30
	Mbandaka	XT	6
	Paratyphi B var. Java	XT	40
	Typhimurium	XT	20
	Typhimurium monophasic (1,4,[5],12:i:−)	XT	17
SeqSero and/or SeqSero2 failed to detect O antigen	Agona	XT	5
	Bareilly	XT	3
	Enteritidis	XT	2
	Infantis	XT	40
	Mbandaka	XT	24
	Paratyphi B var. Java	XT	13
	Typhimurium	XT	10
	Typhimurium monophasic (1,4,[5],12:i:−)	XT	7
	Virchow	XT	3

a Flex kit, Nextera DNA Flex library preparation kit; XT kit, Nextera XT DNA library preparation kit, both Illumina.

b Numbers of isolates of S. enterica serovars are as follows: Agona (*n* = 68), Bareilly (*n* = 5), Enteritidis (*n* = 362), Infantis (*n* = 156), Mbandaka (*n* = 62), Paratyphi B var. Java (*n* = 130), Virchow (*n* = 3), Typhimurium (*n* = 213), and Typhimurium monophasic (1,4,[5],12:i:−) (*n* = 192).

c The SISTR O antigen detection warning is as follows: “*Wzx/Wzy* genes missing. Cannot determine O antigen group/serogroup. Cannot accurately predict serovar from antigen genes.”

**FIG 3:**
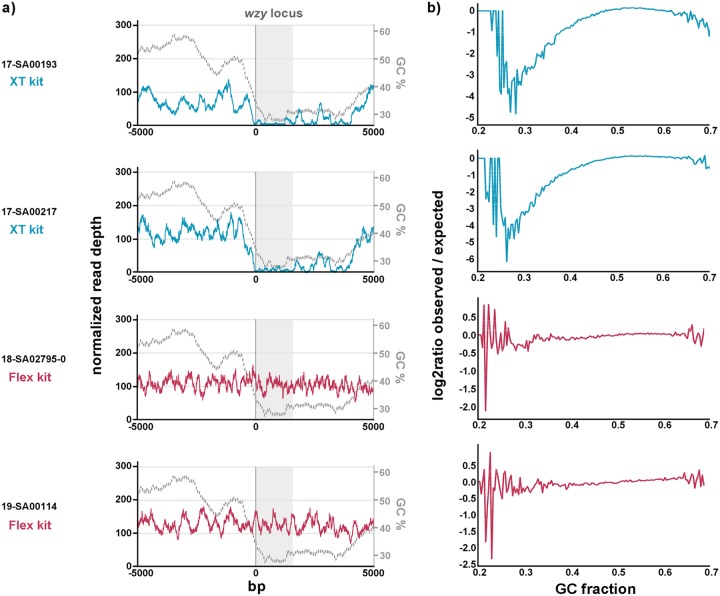
(a) Correlation between GC content and read depth across the *wzy* locus. The colored line graphs (left *y* axis) display the read depths of four serovar Infantis isolates mapped against a reference genome (strain NCTC6703; NCBI accession number NZ_LS483479.1). The gray dashed line (right *y* axis) displays the GC content graph of the reference genome (the GC content was calculated with a perl script available from https://github.com/DamienFr/GC-content-in-sliding-window- with a step size of 1 nucleotide). The position of the *wzy* gene is highlighted with a gray box and was determined through BLAST of the reference genome against the SeqSero2 O antigen database (best match, O-7_wzy_1080: 99.8% identity, 1,080 bp in length, 100% coverage, 2 mismatches). (b) The graph shows the normalized observed/expected read counts per 300 bp across the whole genome. The GC bias was calculated using Benjamini’s method (17) with help from the computeGCBias function of the deepTools package (18). The function counts the number of reads per GC fraction and compares them to the expected GC profile, calculated by counting the number of DNA fragments per GC fraction in a reference genome. In an ideal experiment, the observed GC profile would match the expected profile, producing a flat line at 0. The fluctuations on the ends of the *x* axis are due to the fact that only a small number of genome regions have extreme GC fractions, so that the number of fragments that are picked up in the random sampling can vary. The library kits with which the respective isolates were sequenced are indicated by line colors in both figures (blue, Nextera XT DNA library preparation kit; red, Nextera DNA Flex library preparation kit [both Illumina]).

Sample preparation for whole-genome sequencing assumes that genomic DNA break points are random and sequence independent to produce overlapping fragments. Unfortunately, enzyme-based DNA fragmentation often features sequence biases. According to Illumina, the producer of both library preparation kits used in this study, the enzymatic chemistry of the Flex kit is very different from that of the XT kit. Therefore, it is likely that there is a bias introduced into the sequencing libraries by the enzymes used in the XT kit, which were later modified in the Flex kit. We suspect that the bias is caused by base composition. Both the *wzx* and the *wzy* gene sequence have a considerably lower GC content (∼29.5%) than the average *Salmonella* species open reading frame (∼52.6%). To test our hypothesis, we mapped the reads of four Infantis isolates (two of which were sequenced with the XT kit and two with the Flex kit) against a reference genome and correlated the normalized read depth against the GC content. We observed that across regions with low GC content, such as the *wzy* locus, the normalized read depth was decreased for isolates sequenced with the XT kit, while no direct correlation was apparent for those sequenced with the Flex kit, as shown by the results in Fig. 3a. To verify this effect across the whole genome, we calculated the global GC bias using Benjamini’s method (17) with the deepTools computeGCBias function (18). The results presented in Fig. 3b show that the sequencing data of isolates processed with the XT kit have a strong GC bias, while no similar GC bias is apparent for those sequenced with the Flex kit. Overall, from our results, it is clear that the use of the Flex kit is favorable for O antigen detection.

### Nomenclature.

There were some major differences in how the different tools employed serovar names, which were standardized prior to the analysis to avoid false-negative results. For example, SISTR denoted S. enterica serovar Typhimurium monophasic consistently with its seroformula I 4,5,12:i:−, while SeqSero and SeqSero2 used the term “potential monophasic variant of Typhimurium.” Furthermore, MOST designated S. enterica serovar Goldcoast as “Gold-Coast” and S. enterica serovar Bovismorbificans as “Bovis-Morbificans.” In addition, SeqSero employed the seroformula 61:k,1,5 for S. enterica serovar IIIb 61:k:1,5,(7). This clearly points to the fact that the underlying databases need to be improved and updated.

### Computing time.

We allocated one central processing unit (CPU) on a high-performance computer with 2 terabytes (Tb) of random access memory (RAM) to the serotype predictions and measured the time necessary for computing the serotype results for a representative selection of 5 isolates (S. enterica serovars Agona, Enteritidis, Infantis, Bovismorbificans, and IIIb 61:k:1,5,7). The results are shown in Table S5. The different tools varied greatly in the time they took for calculations. SeqSero2 in k-mer mode was by far the fastest tool, which on average only took ∼10 s per isolate. Also very fast in terms of computing time was SISTR, which generally generated a serovar prediction result in under 30 s (not taking into account the time needed to generate the genome assembly). SeqSero2 in allele mode completed computations in under 4 min, similarly to SeqSero, which was only slightly faster. MOST had the longest calculation time and required ∼6 min per isolate on average.

### Retesting results confirm *in silico* serotyping predictions.

Sixteen isolates for which the *in silico* serovar prediction varied from the laboratory serological test result were retested to verify the correctness of either the experimental or the computed result. Results for laboratory retesting are found in Table S6. In most cases, laboratory retesting confirmed the *in silico* serotyping result, while remaining cases could be traced to mislabeled isolate names, mixed cultures, and transcription and database entry errors.

## DISCUSSION

We determined *Salmonella* serotypes directly from short reads and assembled draft genomes of 1,624 isolates using four different command-line *in silico* tools and compared the serotype prediction results to those of laboratory serotyping performed by slide agglutination. We found that any of the four *in silico* serovar prediction tools tested was able to correctly and unambiguously determine the serovar of >79% of the sample set. Overall, the Salmonella *in silico* Typing Resource (SISTR) produced the most accurate and reliable serotyping results, correctly predicting the serovars of 94% of all isolates, followed by SeqSero2 (87%), SeqSero (81%), and MOST (79%). Furthermore, all *in silico* tools tested were able to determine the serovars of isolates that were nontypeable with laboratory serological testing.

In comparison to other studies, we observed fewer correct matches for MOST than did Ashton et al. (12), and we obtained fewer correct serovar predictions for SeqSero and SeqSero2 than reported by Zhang and colleagues (3, 4). Interestingly, we obtained more accurate results from SeqSero2 in k-mer mode than from the allele microassembly mode, while Zhang and colleagues attribute a higher robustness to the microassembly workflow of SeqSero2.

For SISTR, our study and that of Yoshida et al. (5) show similar numbers of correct serovar predictions. Compared to the independent ENGAGE report (16), we observed more correct serovar predictions for SISTR and SeqSero and fewer accurate results for MOST. Naturally, serotyping comparisons are highly dependent on the compositions of the respective sample sets, and none of the isolates from the ENGAGE study were submitted for retesting, leaving the question open whether the original laboratory serotyping result was correct. Another possible explanation for divergent results is that sample sets used in different studies may have a geographical bias, since the prevalence of certain serovars varies by geographical region. For example, serovars common to North America (e.g., S. enterica serovars Montevideo and Kentucky) were underrepresented in the current study, while serovars common to Europe (e.g., S. enterica serovars Dublin and Senftenberg) were overrepresented (19).

As shown in the 22nd EURL-Salmonella interlaboratory comparison study (20), 35 national *Salmonella* reference laboratories were able to correctly determine the serotypes of 98% of a diverse set of 20 *Salmonella* isolates through serological testing. Therefore, the accuracy of *in silico* serotyping is still slightly behind that of laboratory serotyping results, although SISTR achieved a comparably high percentage of correctly typed isolates (94%).

When comparing the different *in silico* serotyping methodologies, we found that although SISTR cgMLST and SISTR mash generally yielded more accurate and reliable serovar predictions than antigen-mapping-based methods as employed by SeqSero, SeqSero2, and SISTR antigen, antigen mapping was more reliable for serovars with monophasic variations. For example, both SeqSero and SeqSero2 demonstrated greater discriminatory power than SISTR and MOST in differentiating S. enterica serovar Typhimurium and its monophasic variants, for the reason that sequence types that contain two or more serovars do not allow an unambiguous serovar assignment. Another often discussed disadvantage of inferring serovar predictions from sequencing types is that for isolates with a novel sequence type, no associated serovars are available in the database and, therefore, no prediction can be obtained. For example, our sample set of 1,624 isolates contained 15 isolates with novel Achtman 7-gene sequence types, for which MOST was unable to determine a serovar.

On the other hand, mapping-based methods often struggle with closely related serovars that share the same antigenic seroformula but feature minor differences in their O antigenic factors, such as S. enterica serovar Kottbus or Ferruch, as is apparent from the increased number of inconclusive matches that SeqSero, SeqSero2, and SISTR antigen produced.

On a positive note, SISTR combines the advantages of cgMLST and individual antigen detection, making it the most powerful and robust tool in our study. Interestingly, the much simpler algorithm employed by SeqSero2 in k-mer mode was statistically just as successful as the more complex SISTR application. Combined with the advantage that the SeqSero2 k-mer mode does not require an assembly step, thus reducing the effect of genome assembly quality on serovar prediction, and that it had the fastest overall computing time, this makes SeqSero2 in k-mer mode a viable and interesting alternative to SISTR.

Finally, our study clearly demonstrates that variations in library preparation can have significant effects on serotyping results, based on the potential of introducing GC bias during the fragmentation step and subsequent size selection, which lead to depleted reads for important antigen sequences. A known issue of mapping-based tools for the recognition of the *rfb* cluster was solved through the use of the Nextera Flex DNA library preparation kit.

Together, our results indicate that any of the four tools tested would be suitable for replacing traditional laboratory serotyping, which is time and resource consuming, labor intensive, and can be limited by the nonexpression of surface antigens. In contrast, *in silico* serotyping is a rapid, efficient, inexpensive, and reproducible analysis process. Command-line tools, such as those investigated in this study, can be integrated into in-house analysis pipelines, automating serotyping and characterization analysis and reducing potential errors introduced through sample labeling and manual transcriptions.

One of the biggest drawbacks of *in silico* serotyping, the quality of the underlying databases, will readily improve with larger and better curated data sets. This will also improve the serovar prediction for the classification of S. enterica subspecies II to IV, which is still a challenge, both for mapping-based and whole-genome-based methods, for the reason that serovars from subspecies other than S. enterica subsp. *enterica* are not well represented in the databases. Additionally, we would like to encourage the standardization of serovar names for easy comparison of serovar predictions, perhaps through ontology projects, such as GenEpiO and FoodOn (21, 22).

In conclusion, based on the results from our study, we plan to replace traditional serotyping with serovar information obtained through WGS in our laboratory in the near future. Finally, we deposited the complete sample set of this study into the NCBI database, together with the verified serovar information, thus contributing valuable data to the public repositories. We hope that, together with the data generated in previous studies, our released sample set will contribute to the improvement of the underlying databases needed for accurate *in silico Salmonella* serovar prediction now and in the future.

## MATERIALS AND METHODS

### Study design.

Illumina short-read sequencing data of 1,624 *Salmonella* isolates was used to evaluate the performance of four *in silico Salmonella* serotype prediction tools: SeqSero, SeqSero2, SISTR, and MOST. Serovar names were standardized through manual curation, and results were compared to laboratory serotyping results and categorized as full, inconclusive, incongruent, or incorrect matches in keeping with the methodology of Yachison and colleagues (15), as shown in Table 4. Comparison and statistical evaluation of the results were conducted with a customized R script.

**TABLE 4:** Categories for classifying results as full, inconclusive, incongruent, or incorrect matches, in keeping with the methodology of Yachison and colleagues (15)

Match category	Description	Example of result(s) by:	
		Laboratory serotyping	*In silico* prediction
Full	Serovar prediction concordant with traditional typing result	Agona	Agona
Inconclusive	Partial/incomplete serovar prediction or several possible serovars indicated	Senftenberg	Senftenberg or Dessau
Incongruent	Serovar prediction incongruent with traditional typing results due to antigen genes not being expressed	Rough, nonmotile	Paratyphi B var. Java
Incorrect	Serovar prediction incorrect with respect to traditional typing results	Typhimurium	Infantis
No result	*In silico* tool did not produce a serotype prediction, i.e., serovar name or seroformula	Mbandaka	None

### Study isolates.

**FIG 4:**
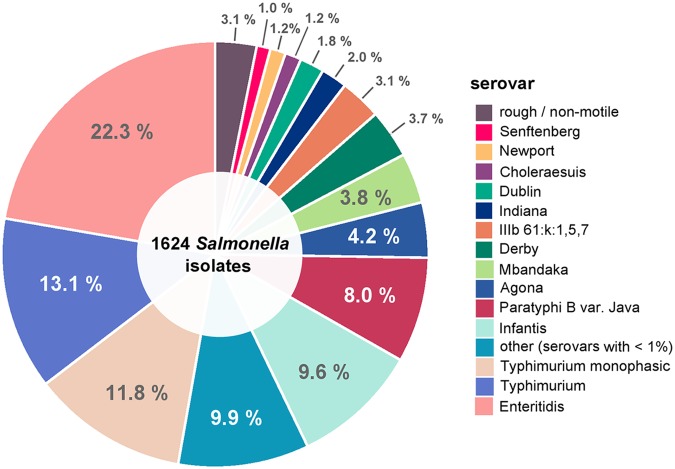
Graphical representation of the composition of the sample set by serotypes. The pie chart shows the composition of the analyzed sample set of 1,624 isolates grouped by serotypes as determined through serological testing. Isolates that could not be typed in the laboratory are listed in the category “rough/nonmotile.”

1,624 *Salmonella* isolates that were submitted to the German National Salmonella Reference Laboratory were included in this analysis. The National Reference Laboratory receives, on average, 3,500 to 4,000 *Salmonella* species isolates from nonhuman sources for routine analysis yearly. Of those, about 10 to 20% are chosen for whole-genome short-read sequencing as part of outbreak investigations and for additional study purposes. Therefore, the isolates included in this study realistically represent serovars from both animals and foodstuffs submitted to the Reference Laboratory between the years 1999 and 2019. Consequently, the resulting sample set of 1,624 Salmonella enterica isolates is biased toward the most commonly occurring, public health-relevant foodborne serovars occurring in Germany, as shown in Fig. 4 (a full list of included serovars is given in Table S2 in the supplemental material). Almost half of the complete sample set is made up of S. enterica serovar Typhimurium, together with its monophasic variation (∼25%), and S. enterica serovar Enteritidis (∼22%). S. enterica serovars Infantis (∼10%) and Paratyphi B var. Java (∼8%) are also well represented within the sample set, with over 100 isolates each. About 60 isolates each belong to S. enterica serovars Agona (4.2%), Mbandaka (3.8%), and Derby (3.7%). Less frequently encountered serovars, such as S. enterica serovars Indiana, Dublin, Choleraesuis, Newport, Senftenberg, Coeln, Saintpaul, and Orion (each with 10 or more isolates), make up about 10% of the sample set. The remainder of the sample set (∼10%) contains rarely encountered serovars with just a small number or an individual isolate each. Among these are the S. enterica serovars Agama, Ohio, Bareilly, Hadar, Kottbus, Mikawasima, Montevideo, Napoli, Schwarzengrund, Stourbridge, Brandenburg, Heidelberg, Livingstone, Rissen, Virchow, Bovismorbificans, Corvallis, Give, Oranienburg, Anatum, Blockley, Bredeney, Glostrup, Hessarek, Idikan, Kedougou, Kentucky, and Wagenia. Furthermore, 56 isolates from S. enterica subsp. IIIb, as well as individual isolates from S. enterica subspecies II, IIIa, and IV and one Salmonella bongori isolate, were included in the analysis. In addition, isolates which could not be typed in the laboratory were included as rough or nonmotile serovars (∼3%).

### Laboratory serotyping.

All isolates used in this study were routinely serotyped according to the White-Kauffmann-Le Minor scheme (23) by slide agglutination with O and H antigen-specific sera (Sifin Diagnostics, Berlin, Germany). From 2015 onwards, all isolates serotyped as I 4,[5],12:i:− were additionally confirmed as S. enterica serovar Typhimurium monophasic by real-time PCR (24).

### Whole-genome sequencing and assembly.

Bacteria were cultivated on LB agar. A single colony was inoculated into liquid LB and cultivated under shaking conditions (180 to 220 rpm) at 37°C for 14 to 16 h. Genomic DNA was extracted from liquid cultures using the PureLink genomic DNA minikit (Invitrogen, Carlsbad, CA, USA). Sequencing libraries were prepared with the Nextera XT DNA library preparation kit or the Nextera DNA Flex library preparation kit (Illumina, San Diego, CA, USA) according to the manufacturer’s protocol. Paired-end sequencing was performed on the Illumina MiSeq benchtop sequencer using the MiSeq reagent kit version 3 (600 cycle) or on the Illumina NextSeq 500 benchtop sequencer using the NextSeq 500/550 midoutput kit version 2 or version 2.5 (300 cycle). Raw reads were trimmed using fastp version 0.19.5 (25) and *de novo* assembled using unicycler version 0.4.4 (26).

### *In silico* serotyping.

Descriptions of tools and parameters used to obtain the serotype prediction results are given in Table S1.

### Data availability.

Sequencing data for all isolates analyzed in this study have been deposited in the NCBI Sequence Read Archive (SRA) under BioProject accession numbers PRJEB31846, PRJEB23094, PRJEB30118, PRJEB16326, and PRJEB30493.

## Supplementary Material

Supplemental file 1
