# Supplementary material for: Performance and Accuracy of Four Open-Source Tools for In Silico Serotyping of Salmonella spp. Based on Whole-Genome Short-Read Sequencing Data
Source: Appl Environ Microbiol. 2020 Feb 18;86(5):e02265-19. doi: 10.1128/AEM.02265-19 (PMC7028957; doi:10.1128/AEM.02265-19)
Supplement: Supplemental file 1 [file AEM.02265-19-s0001.pdf]

Supplemental Material

Table S1: Description of tools and parameters used to obtain serotype prediction results.

**SeqSero v1.0.0 stand-alone, command line tool**		
Mode	default parameters	SeqSero is a tool for serotype prediction, which analyses the sequences of the *Salmonella* O- and H-antigen determinants. It is based on a curated databases of nucleotide sequences for the O-antigen (*wzx* flippase gene, *wzy* polymerase gene, additional genes from the *rfb* cluster) and the two H-antigens (*fliC* gene, *fljB* gene). SeqSero takes paired raw reads as input and maps these raw reads using the Burrows-Wheeler Aligner (BWA) against the database. It then counts how many reads map to which allele and chooses the allele with the highest number of mapped reads as respective for the isolate. Finally, it assigns the serovar from the code combination of the highest scoring alleles according to the White-Kauffmann-LeMinor scheme.
Input	raw short read sequences	
Source	https://github.com/denglab/SeqSero	
Reference	(1)	
Webtool version	http://denglab.info/SeqSero	

**SeqSero2 v1.0.0 stand-alone, command line tool**		
Mode	default parameters, allele micro-assembly mode and k-mer mode	SeqSero2 is an updated version of SeqSero with a refined approach for serotype prediction. In the default mode SeqSero2 takes raw reads as input and performs targeted assembly of serotype determinant alleles with SPAdes. Assembled alleles are then mapped to a curated database with the Burrows-Wheeler Aligner and serotypes are predicted on the best match. A big advantage of this method is that potential contamination in sequencing data (i.e. presence of reads from multiple serotypes resulting from cross or carry-over contamination during sequencing) can be detected. In a second workflow, SeqSero2 takes raw reads or assembled genomes as input and perform rapid serotype prediction based on unique k-mers of serotype determinants.
Input	raw short read sequences, draft genome assemblies	
Source	https://github.com/denglab/SeqSero2	
Reference	(2)	
Webtool version	none implemented	

**SISTR_cmd v1.0.2 stand-alone, command line tool**		
Mode	default parameters	*Salmonella in silico* Typing Resource (SISTR**)** takes draft *Salmonella* genome assemblies as input. It then proceeds similarly to SeqSero, in that it compares the sequences of the *wzx* and *wzy* genes, and the *fliC* and *fljB* genes to a curated database. Furthermore, SISTR is able to resolve ambiguous serotypes with cgMLST (330 locus core genome MLST). To do so, SISTR clusters the isolates with cgMLST and then assign the most likely serotype based on the phylogenetic clustering
Input	draft genome assemblies	
Source	https://github.com/phac-nml/SISTR_cmd	
Reference	(3)	
Webtool version	https://lfz.corefacility.ca/sistr-app/	

		of any individual isolate within groups of serovars. SISTR combines the output of these two different methods and provides an overall serotype prediction result. SISTR produces four different serovar prediction results. Serovar_antigen is the prediction from the O- and H-antigen sequence matching alone. Serovar_cgmlst is the prediction from cgMLST similarity to the closest matching reference genome. *Serovar* is the overall prediction from both the antigen search and cgMLST results. The cgMLST prediction overrides the antigen prediction in the case of a high similarity match to a reference genome (cgMLST distance <= 0.05). cgMLST is also used to predict the subspecies and the general quality of the genome sequencing data, based on the presence /absence of core genes. The final serovar result is an aggregate of the O- and H-antigen prediction and the cgMLST (or Mash) prediction.

MOST v1.0 stand-alone, command line tool

Mode	default parameters	Metric-Oriented Sequence Typer (MOST) is a modified version of SRST (version 1) (4). It uses short read Illumina reads to call MLST profiles, which it then uses to infer serovar predictions. In short, it first uses the bowtie2 alignment algorithm to map paired raw reads to a sequence database of all MLST allele sequences and then determines the allele at each locus. Only when reads can be mapped with zero SNPs or indels, the allele is chosen. If all alleles could be found MOST assigns the ST value. Finally, the program searches (in keeping with its acronym) for the most common serovar with this ST value in the PHE/Achtmann database and outputs a list with the number of the reported ST – serovar combinations.
Input	raw short read sequences	
Source	https://github.com/phe-bioinformatics/MOST	
Reference	(5)	
Webtool version	none implemented	

Table S2: Overview of tool performance in percentages grouped by serovar.

serovar	n	match	SeqSero	SeqSero2 allele	SeqSero2 k-mer	SISTR overall	SISTR antigen	SISTR cgMLST	SISTR mash	MOST
Agama	6	full	100	100	100	100	100	66.67	66.67	83.33
		inconclusive								
		incorrect						33.33	33.33	
		no result								16.67
		incongruent								
Agona	68	full	100	92.65	100	100		100	100	100
		inconclusive					100			
		incorrect		7.35						
		no result								
		incongruent								
Anatum	1	full	100	100	100	100		100	100	100
		inconclusive					100			
		incorrect								
		no result								
		incongruent								
Bareilly	5	full	40	40	60	100		100	100	100
		inconclusive					100			
		incorrect	60	60	40					
		no result								
		incongruent								
Blockley	1	full		100	100	100		100	100	100
		inconclusive	100				100			
		incorrect								
		no result								
		incongruent								
Bovismorbificans	2	full		100	100	100		100	100	100
		inconclusive	100				100			
		incorrect								
		no result								
		incongruent								
Brandenburg	3	full	100	100	100	100	100	100	100	100
		inconclusive								
		incorrect								
		no result								
		incongruent								
Bredeney	1	full	100	100	100	100	100	100	100	100
		inconclusive								
		incorrect								
		no result								
		incongruent								
Choleraesuis	20	full				100		100	100	100
		inconclusive	100	100	100		100			
		incorrect								
		no result								
		incongruent								
Coeln	11	full	100	100	100	100		100	100	100
		inconclusive								
		incorrect					100			
		no result								
		incongruent								
Corvallis	1	full				100		100	100	100
		inconclusive	100	100	100		100			
		incorrect								
		no result								
		incongruent								
Derby	60	full	96.67	85	96.67	100		100	100	100
		inconclusive					100			
		incorrect	3.33	15	3.33					
		no result								
		incongruent								
Dublin	30	full	96.67	93.33	73.33	100		100	100	100
		inconclusive					90			
		incorrect	3.33	6.67	26.67		10			
		no result								
		incongruent								
Enteritidis	362	full	90.88	91.16	84.81	99.72		99.72	99.72	98.62

		inconclusive	8.84	8.29	7.73		95.58			
		incorrect	0.28	0.55	7.46	0.28	4.42	0.28	0.28	
		no result								1.38
		incongruent								
Give	2	full	100	100	100	100		100	100	100
		inconclusive					100			
		incorrect								
		no result								
		incongruent								
Glostrup	1	full				100		100	100	100
		inconclusive	100	100	100		100			
		incorrect								
		no result								
		incongruent								
Goldcoast	3	full				100		100	100	100
		inconclusive	100	100	100		100			
		incorrect								
		no result								
		incongruent								
Hadar	7	full		100	100	100		100	100	100
		inconclusive	100				100			
		incorrect								
		no result								
		incongruent								
Havana	4	full	100	100	100	100	100	100	75	75
		inconclusive								
		incorrect							25	
		no result								25
		incongruent								
Heidelberg	4	full	100	100	100	100	100	100	100	100
		inconclusive								
		incorrect								
		no result								
		incongruent								
Hessarek	1	full				100	100			100
		inconclusive	100	100	100					
		incorrect						100	100	
		no result								
		incongruent								
I 11:z41:e,n,z15	6	full			83.33					
		inconclusive	66.67							
		incorrect	33.33	100	16.67	100	100	100	100	100
		no result								
		incongruent								
I 4,12:d:-	8	full								
		inconclusive								
		incorrect	100	100	100	100	100	100	100	
		no result								100
		incongruent								
Idikan	1	full	100	100	100	100		100	100	100
		inconclusive					100			
		incorrect								
		no result								
		incongruent								
IIIa 41:z4,z23:-	1	full		100	100	100		100	100	
		inconclusive	100				100			
		incorrect								100
		no result								
		incongruent								
IIIb 61:k:1,5,7	51	full		100	100	100	78.43	98.04	98.04	
		inconclusive	100							
		incorrect					21.57	1.96	1.96	98.04
		no result								1.96
		incongruent								
IIIb 61:l,v:1,5,7	1	full		100	100					
		inconclusive	100			100	100	100	100	
		incorrect								100
		no result								
		incongruent								
IIIb 61:l,v:z35	2	full	100	100	100	100	100	100		
		inconclusive								

		incorrect							100	
		no result								100
		incongruent								
IIIb 61:z52:z53	2	full	100	100	100	100	100	100	100	
		inconclusive								
		incorrect								100
		no result								
		incongruent								
Indiana	32	full		100	100	100	100	100	100	100
		inconclusive	100							
		incorrect								
		no result								
		incongruent								
Infantis	156	full	89.74	75	94.87	100		100	100	100
		inconclusive					100			
		incorrect	10.26	25	5.13					
		no result								
		incongruent								
Isangi	1	full	100	100	100	100		100	100	100
		inconclusive					100			
		incorrect								
		no result								
		incongruent								
Kedougou	1	full	100	100	100	100	100	100	100	100
		inconclusive								
		incorrect								
		no result								
		incongruent								
Kentucky	1	full	100	100	100	100	100	100	100	100
		inconclusive								
		incorrect								
		no result								
		incongruent								
Kiambu	2	full		100	100	100	100	100	100	100
		inconclusive	100							
		incorrect								
		no result								
		incongruent								
Kottbus	5	full				100		100	100	100
		inconclusive	100	100	100		100			
		incorrect								
		no result								
		incongruent								
Liverpool	1	full	100	100	100	100		100	100	100
		inconclusive					100			
		incorrect								
		no result								
		incongruent								
Livingstone	6	full	83.33	83.33	83.33	100	83.33	100	100	50
		inconclusive					16.67			
		incorrect	16.67	16.67	16.67					50
		no result								
		incongruent								
Martonos	1	full				100		100	100	
		inconclusive	100	100	100		100			
		incorrect								
		no result								100
		incongruent								
Mbandaka	62	full	72.58	67.74	91.94	100	64.52	100	100	98.39
		inconclusive					33.87			
		incorrect	27.42	32.26	8.06		1.61			
		no result								1.61
		incongruent								
Mikawasima	5	full	100	80	100	40	20	100	100	40
		inconclusive					20			
		incorrect		20		60	60			
		no result								60
		incongruent								
Montevideo	8	full	100	100	100	100		100	100	100
		inconclusive					100			
		incorrect								

		no result								
		incongruent								
rough / non-motile	51	full								
		inconclusive								
		incorrect								
		no result								
		incongruent	100	100	100	100	100	100	100	100
Napoli	8	full	100	100	87.5	87.5	87.5	87.5	100	50
		inconclusive								
		incorrect			12.5	12.5	12.5	12.5		50
		no result								
		incongruent								
Newport	19	full	100	100	100	100		100	100	94.74
		inconclusive					100			
		incorrect								
		no result								5.26
		incongruent								
Ohio	5	full	100	100	100	100	100	100	100	100
		inconclusive								
		incorrect								
		no result								
		incongruent								
Oranienburg	2	full		100	100	100		100	100	100
		inconclusive	100				100			
		incorrect								
		no result								
		incongruent								
Orion	10	full	100	100	100	100		100	100	100
		inconclusive								
		incorrect					100			
		no result								
		incongruent								
Paratyphi B var. Java	130	full	96.92	91.54	98.46	100		100	100	94.62
		inconclusive					98.46			
		incorrect	3.08	8.46	1.54		1.54			
		no result								5.38
		incongruent								
Paratyphi B var. Java monophasic	2	full	100	100	100					
		inconclusive								
		incorrect				100	100	100	100	100
		no result								
		incongruent								
Putten	1	full	100	100	100	100	100	100	100	100
		inconclusive								
		incorrect								
		no result								
		incongruent								
Rissen	3	full	100	100	100	100	100	100	100	100
		inconclusive								
		incorrect								
		no result								
		incongruent								
Saintpaul	11	full	100	100	100	100	100	100	100	100
		inconclusive								
		incorrect								
		no result								
		incongruent								
Schwarzengrund	4	full	100	100	100	100	100	100	100	100
		inconclusive								
		incorrect								
		no result								
		incongruent								
Senftenberg	17	full		11.76		100	94.12	100	100	100
		inconclusive	100	88.24	100		5.88			
		incorrect								
		no result								
		incongruent								
Stourbridge	1	full	100	100	100	100	100	100	100	
		inconclusive								
		incorrect								
		no result								100

		incongruent								
Tennessee	1	full		100	100	100	100	100	100	100
		inconclusive	100							
		incorrect								
		no result								
		incongruent								
Thompson	1	full	100	100	100	100		100	100	100
		inconclusive					100			
		incorrect								
		no result								
		incongruent								
Typhimurium	213	full	99.53	94.84	99.53	93.9	88.26	93.9	95.77	99.53
		inconclusive	0.47	0.47	0.47	6.1	11.74	6.1	4.23	
		incorrect		4.69						
		no result								0.47
		incongruent								
Typhimurium monophasic	192	full	90.1	79.17	91.67	96.88	91.67	96.88	91.15	
		inconclusive	9.38	8.85	8.33	3.12	7.81	3.12	8.85	98.44
		incorrect	0.52	11.98			0.52			
		no result								1.56
		incongruent								
V 11:z41:e,n,x	1	full								
		inconclusive								
		incorrect	100	100	100			100	100	
		no result				100	100			100
		incongruent								
V 60:z41:-	1	full				100	100	100	100	100
		inconclusive		100						
		incorrect	100		100					
		no result								
		incongruent								
Virchow	3	full	33.33		66.67	100		100	100	100
		inconclusive					100			
		incorrect	66.67	100	33.33					
		no result								
		incongruent								
Wagenia	2	full	100	100	100	100	100			
		inconclusive								
		incorrect						100	100	
		no result								100
		incongruent								

Table S3: Examples for serovar prediction for isolates from subspecies II to V.

Isolate	Laboratory	SeqSero	SeqSero2 k-mer	SeqSero2 allele	SISTR	SISTR antigen	SISTR cgMLST	SISTR mash	MOST	SISTR cgMLST subspecies
19-SA01113-0	rough / non-motile	7:z10:z39	II -:l,z13,z28:z39	II 7:l,z13,z28:z39	II 6,7:l,z13,z28:1,6	II 6,7:l,z13,z28:1,6	II 58:d:z8	II 53:l,z28:z39	no result	salamae
17-SA01395-0	rough / non-motile	II 4,12:l,w:e,n,x	II 4,12:l,w:e,n,x	II 4,12:l,w:e,n,x	II 4,12:l,w:e,n,x	II 4,12:l,w:e,n,x	II 9,46:l,w:e,n,x	II 9,46:l,w:e,n,x	no result	salamae
08-03244	V 11:z41:e,n,x	11:z6:k	VIII 11:z6:e,n,x,z15	II -:z41:e,n,x,z15	no result	no result	II 55:z39:-	II 41:b:-	no result	salamae
17-SA03206-0	IIIa 41:z4,z23:-	Waycross or IV 41:z4,z23:- or IIIa 41:z4,z23:-	IIIa 41:z4,z23:-	IIIa 41:z4,z23:-	IIIa 41:z4,z23:-	IIIa 41:z4,z23:- or IIIa 62:z4,z23:-	IIIa 41:z4,z23:-	IIIa 41:z4,z23:-	arizonae	arizonae
18-SA03929-0-S2	rough / non-motile	Zinder or IV 44:z29:-	IIIa -:z29:-	Zinder or IV 44:z29:-	IIIa 44:z29:-	IIIa 44:z29:-	IIIa 18:z4,z32:-	IIIa 18:z4,z32:-	no result	arizonae
14-SA00608	IIIb 61:l,v:z35	IIIb 61:l,v:z35	IIIb 61:l,v:z35	IIIb 61:l,v:z35	IIIb 61:l,v:z35	IIIb 61:l,v:z35	IIIb 61:l,v:z35	IIIb 47:k:z35	no result	diarizonae
11-03263	IIIb 61:z52:z53	IIIb 61:z52:z53	IIIb 61:z52:z53	IIIb 61:z52:z53	IIIb 61:z52:z53	IIIb 61:z52:z53	IIIb 61:z52:z53	IIIb 61:z52:z53	arizonae	diarizonae
19-SA00884-0	rough / non-motile	58:z10:z	IIIb 58:z10:z	IIIa 58:z10:z	IIIb 58:z10:z	IIIb 58:z10:z	IIIb 50:r:z:z67	IIIb 47:k:z35	no result	diarizonae
18-SA01611-S2	rough / non-motile	IV 48:g,z51:- or IIIa 48:g,z51:-	IV 48:g,z51:-	IV 48:g,z51:-	IV 48:g,z51:-	IV 48:g,z51:-	IV 1,44:z4,z32:-	IV 50:z4,z23:-	no result	houtenae
10-00679-0	V 60:z41:-	-:z41:-	V -:z41:-	60:z41:-	V 60:z41:-	V 60:z41:-	V 60:z41:-	V 60:z41:-	V 60:z41:-	bongori

Table S4: A Fisher's exact test was used to evaluate the statistical significance between the successful predictions of each tool. The Fisher's exact test was calculated with the *fisher.multcomp* function from the R Package 'RVAideMemoire' (https://cran.r-project.org/package=RVAideMemoire).

p-value (pairwise comparisons using Fisher's exact test)	SISTR	SISTR mash	SISTR antigen	SISTR cgMLST	SeqSero	SeqSero2 k-mer	SeqSero2 allele
**SISTR mash**	1.00e+00						
**SISTR antigen**	1.35e-18	1.35e-18					
**SISTR cgMLST**	1.00e+00	1.00e+00	1.35e-18				
**SeqSero**	1.98e-02	1.98e-02	2.19e-10	1.98e-02			
**SeqSero2 k-mer**	2.16e-01	2.16e-01	1.68e-13	2.16e-01	4.47e-01		
**SeqSero2 allele**	2.67e-02	2.67e-02	8.43e-11	2.67e-02	1.00e+00	5.53e-01	
**MOST**	8.84e-03	8.84e-03	1.62e-09	8.84e-03	1.00e+00	2.62e-01	8.78e-01

Table S5: Time required to calculate serovar predictions for selected isolates.

Isolate	Serovar	SeqSero	SeqSero2 k-mer	SeqSero2 allele	SISTR	MOST
18-SA00897-S2	Agona	1m 55.008s	0m 7.384s	2m 20.836s	0m 28.224s	4m 48.660s
18-SA04233	Enteriditis	2m 59.464s	0m 6.600s	3m 10.656s	0m 27.492s	7m 33.116s
16-SA00403	Infantis	2m 50.552s	0m 8.320s	3m 1.676s	0m 27.956s	6m 21.128s
18-SA02723-1	Bovismorbificans	2m 31.144s	0m 6.840s	2m 33.632s	0m 28.132s	6m 8.112s
16-SA00356	IIIb 61:k:1,5,7	2m 32.364s	0m 7.080s	2m 48.312s	0m 27.868s	6m 22.588s

Table S6: Results for laboratory re-testing

name	laboratory serotyping	SeqSero	SeqSero2 k-mer mode	SeqSero2 allele mode	SISTR	MOST	laboratory retesting result	error type
05-00850	Group B monophasic	Paratyphi B var. Java	Paratyphi B var. Java	Paratyphi B var. Java	Paratyphi B var. Java	Paratyphi B var. Java	Paratyphi B var. Java	transcription error
10-04835	Napoli	Napoli	I 9:l,z13:e,n,x	Napoli	Zaiman	Zaiman	Napoli	no error
11-00369	Napoli	Napoli	Napoli	Napoli	Zaiman	Zaiman	Napoli	no error
11-01173	Typhimurium	Heidelberg	Heidelberg	Heidelberg	Heidelberg	Heidelberg	Heidelberg	erronous serotyping
13-SA00570	Derby	Typhimurium	I 4:f,g:-	Derby	Typhimurium	Typhimurium	Typhimurium	mixed culture
13-SA00570-S2	Derby	Derby	Typhimurium	Typhimurium	Derby	Derby	Derby	mixed culture
13-SA02576	Typhimurium	Paratyphi B var. Java	Paratyphi B var. Java	Paratyphi B var. Java	Paratyphi B var. Java	Paratyphi B var. Java	Paratyphi B var. Java	erronous serotyping
14-SA00512	Infantis	Typhimurium	Typhimurium	Typhimurium	Infantis	Infantis	Typhimurium	mixed culture
15-SA01021-1	Infantis	Agona	Agona	Agona	Agona	Agona	Agona	transcription error
18-SA00308-S2	Goldcoast	Goldcoast or Brikama	Goldcoast or Brikama	Goldcoast or Brikama	Goldcoast	Goldcoast	Goldcoast	no error
18-SA00438-2	Subspecies IIIb	-:g,m:-	I -:g,m:-	I -:g,m:-	Enteritidis	Enteritidis	Enteritidis	erronous serotyping
18-SA01653	Typhimurium	Paratyphi B var. Java	Paratyphi B var. Java	Paratyphi B var. Java	Paratyphi B var. Java	Paratyphi B var. Java	Paratyphi B var. Java	erronous serotyping
18-SA02693-0	Enteritidis	Derby	Derby	Derby	Derby	Derby	Derby	erronous serotyping
18-SA04291-0	Saintpaul	Typhimurium	Typhimurium	Typhimurium	Typhimurium	Typhimurium	Typhimurium	erronous serotyping
19-SA00130-1	Corvallis	Enteritidis	Enteritidis	Enteritidis	Enteritidis	Enteritidis	Enteritidis	transcription error
19-SA00311-0	Paratyphi B var. Java	Typhimurium	Typhimurium	Typhimurium	Typhimurium	Typhimurium	Typhimurium	erronous serotyping

References

1. Zhang S, Yin Y, Jones MB, Zhang Z, Kaiser BLD, Dinsmore BA, Fitzgerald C, Fields PI, Deng X. 2015. *Salmonella* serotype determination utilizing high-throughput genome sequencing data. J Clin Microbiol JCM.00323-15.

2. Zhang S, Den-Bakker HC, Li S, Chen J, Dinsmore BA, Lane C, Lauer AC, Fields PI, Deng X. 2019. SeqSero2: rapid and improved *Salmonella* serotype determination using whole genome sequencing data. Appl Environ Microbiol AEM.01746-19, aem;AEM.01746-19v1.

3. Yoshida CE, Kruczkiewicz P, Laing CR, Lingohr EJ, Gannon VPJ, Nash JHE, Taboada EN. 2016. The Salmonella In Silico Typing Resource (SISTR): An open web-accessible tool for rapidly typing and subtyping draft *Salmonella* genome assemblies. PloS One 11:e0147101.

4. Inouye M, Dashnow H, Raven L-A, Schultz MB, Pope BJ, Tomita T, Zobel J, Holt KE. 2014. SRST2: Rapid genomic surveillance for public health and hospital microbiology labs. Genome Med 6.

5. Tewolde R, Dallman T, Schaefer U, Sheppard CL, Ashton P, Pichon B, Ellington M, Swift C, Green J, Underwood A. 2016. MOST: a modified MLST typing tool based on short read sequencing. PeerJ 4.
